# Supplementary material for: Causal Relationship between Aging and Anorexia Nervosa: A White-Matter-Microstructure-Mediated Mendelian Randomization Analysis
Source: Biomedicines. 2024 Aug 16;12(8):1874. doi: 10.3390/biomedicines12081874 (PMC11351342; doi:10.3390/biomedicines12081874)
Supplement: Supplementary file 1 [file biomedicines-12-01874-s001.zip › Supplementary Figure/Figure S4/Singe SNP plot of MR analysis for TL to ubm-b-1703.pdf]

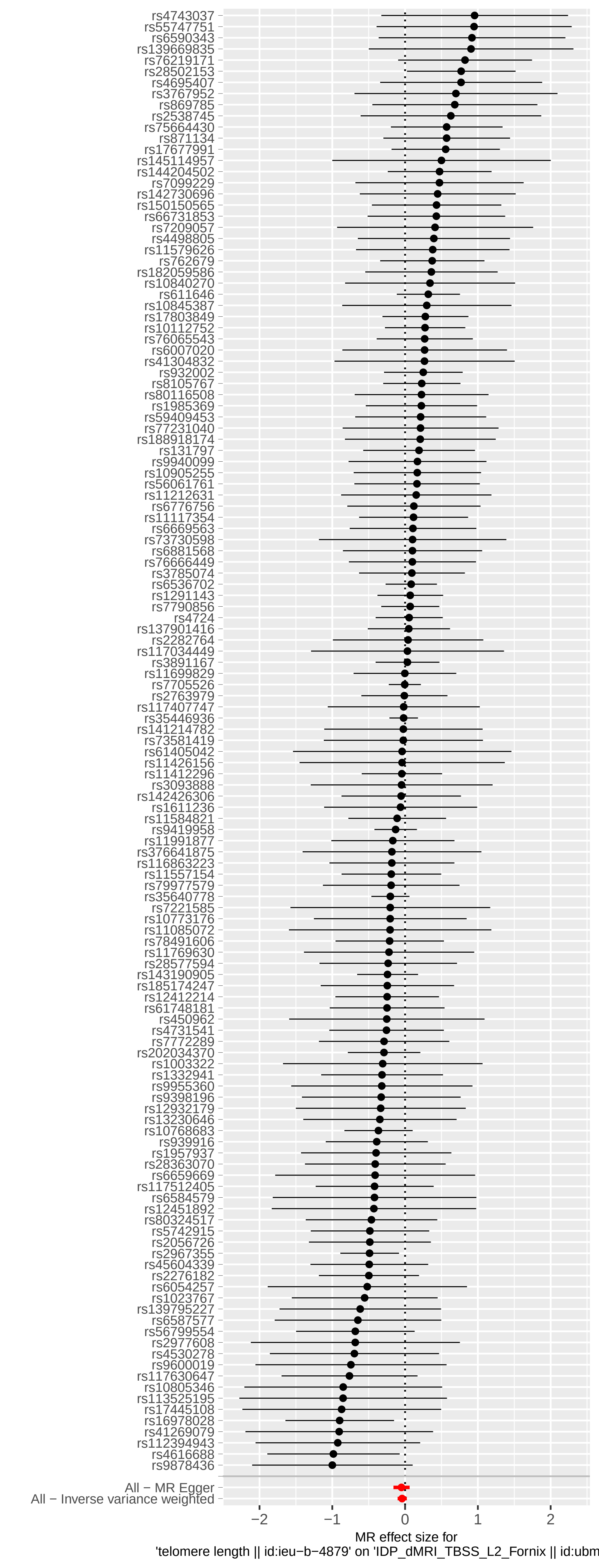
rs4743037
rs55747751
rs6590343
rs139669835
rs76219171
rs28502153
rs4695407
rs3767952
rs869785
rs2538745
rs75664430
rs871134
rs17677991
rs145114957
rs144204502
rs7099229
rs142730696
rs150150565
rs66731853
rs7209057
rs4498805
rs11579626
rs762679
rs182059586
rs10840270
rs611646
rs10845387
rs17803849
rs10112752
rs76065543
rs6007020
rs41304832
rs932002
rs8105767
rs80116508
rs1985369
rs59409453
rs77231040
rs188918174
rs131797
rs9940099
rs10905255
rs56061761
rs11212631
rs6776756
rs11117354
rs6669563
rs73730598
rs6881568
rs76666449
rs3785074
rs6536702
rs1291143
rs7790856
rs4724
rs137901416
rs2282764
rs117034449
rs3891167
rs11699829
rs7705526
rs2763979
rs117407747
rs35446936
rs141214782
rs73581419
rs61405042
rs11426156
rs11412296
rs3093888
rs142426306
rs1611236
rs11584821
rs9419958
rs11991877
rs376641875
rs116863223
rs11557154
rs79977579
rs35640778
rs7221585
rs10773176
rs11085072
rs78491606
rs11769630
rs28577594
rs143190905
rs185174247
rs12412214
rs61748181
rs450962
rs4731541
rs7772289
rs202034370
rs1003322
rs1332941
rs9955360
rs9398196
rs12932179
rs13230646
rs10768683
rs939916
rs1957937
rs28363070
rs6659669
rs117512405
rs6584579
rs12451892
rs80324517
rs5742915
rs2056726
rs2967355
rs45604339
rs2276182
rs6054257
rs1023767
rs139795227
rs6587577
rs56799554
rs2977608
rs4530278
rs9600019
rs117630647
rs10805346
rs113525195
rs17445108
rs16978028
rs41269079
rs112394943
rs4616688
rs9878436
All − MR Egger
All − Inverse variance weighted
−2
−1
0
1
2
MR effect size for
'telomere length || id:ieu−b−4879' on 'IDP_dMRI_TBSS_L2_Fornix || id:ubm
